# Maize/peanut intercropping has greater synergistic effects and home-field advantages than maize/soybean on straw decomposition

**DOI:** 10.3389/fpls.2023.1100842

**Published:** 2023-03-03

**Authors:** Surigaoge Surigaoge, Hao Yang, Ye Su, Yu-He Du, Su-Xian Ren, Dario Fornara, Peter Christie, Wei-Ping Zhang, Long Li

**Affiliations:** ^1^Beijing Key Laboratory of Biodiversity and Organic Farming, College of Resources and Environmental Sciences, China Agricultural University, Beijing, China; ^2^Davines Group-Rodale Institute European Regenerative Organic Center (EROC), Parma, Italy; ^3^Chinese Academy of Sciences (CAS) Key Laboratory of Soil Environment and Pollution Remediation, Institute of Soil Science, Chinese Academy of Sciences, Nanjing, China

**Keywords:** C:N ratio, home-field advantage, litter quality, maize/legume intercropping, mixed litter decomposition, N addition, non-additive effects, plant diversity

## Abstract

**Introduction:**

The decomposition of plant litter mass is responsible for substantial carbon fluxes and remains a key process regulating nutrient cycling in natural and managed ecosystems. Litter decomposition has been addressed in agricultural monoculture systems, but not in intercropping systems, which produce species-diverse litter mass mixtures. The aim here is to quantify how straw type, the soil environment and their combined effects may influence straw decomposition in widely practiced maize/legume intercropping systems.

**Methods:**

Three decomposition experiments were conducted over 341 days within a long-term intercropping field experiment which included two nitrogen (N) addition levels (i.e. no-N and N-addition) and five cropping systems (maize, soybean and peanut monocultures and maize/soybean and maize/peanut intercropping). Experiment I was used to quantify litter quality effects on decomposition; five types of straw (maize, soybean, peanut, maize-soybean and maize-peanut) from two N treatments decomposed in the same maize plot. Experiment II addressed soil environment effects on root decomposition; soybean straw decomposed in different plots (five cropping systems and two N levels). Experiment III addressed ‘home’ decomposition effects whereby litter mass (straw) was remained to decompose in the plot of origin. The contribution of litter and soil effects to the home-field advantages was compared between experiment III (‘home’ plot) and I-II (‘away’ plot).

**Results and discussions:**

Straw type affected litter mass loss in the same soil environment (experiment I) and the mass loss values of maize, soybean, peanut, maize-soybean, and maize-peanut straw were 59, 77, 87, 76, and 78%, respectively. Straw type also affected decomposition in the ‘home’ plot environment (experiment III), with mass loss values of maize, soybean, peanut, maize-soybean and maize-peanut straw of 66, 74, 80, 72, and 76%, respectively. Cropping system did not affect the mass loss of soybean straw (experiment II). Nitrogen-addition significantly increased straw mass loss in experiment III. Decomposition of maize-peanut straw mixtures was enhanced more by ‘home-field advantage’ effects than that of maize-soybean straw mixtures. There was a synergistic mixing effect of maize-peanut and maize-soybean straw mixture decomposition in both 'home' (experiment III) and ‘away’ plots (experiment I). Maize-peanut showed greater synergistic effects than maize-soybean in straw mixture decomposition in their 'home' plot (experiment III). These findings are discussed in terms of their important implications for the management of species-diverse straw in food-production intercropping systems.

## 1 Introduction

Terrestrial plants are estimated to produce 120 Pg of organic carbon (C) annually, and about 60 Pg of this C enters the dead organic matter pool (Datry et al., 2018). Similarly, the amount of crop residue yielded worldwide is estimated at 3758×10^6^ Mg/year for 27 food crops (Lal, 2005). The return of litter mass (i.e. straw) to the soil has been commonly practiced to increase crop yield and manage carbon (C) sequestration in agricultural ecosystems (Liu et al., 2014). Plant litter mass decomposition not only accounts for a substantial carbon (C) flux but is a key process regulating nutrient cycling in terrestrial ecosystems (Barbe et al., 2017; Bichel et al., 2017; Chen et al., 2017a). Plant decomposition has been extensively studied in natural ecosystems and monoculture agricultural ecosystems (Handa et al., 2014; Xu et al., 2017). Intercropping is widely practiced worldwide and is considered a good example of sustainable agriculture (Vandermeer, 1992; Bedoussac et al., 2015; Yang et al., 2017), because it maintains crop yields without increasing inputs (Li et al., 2007; Tamburini et al., 2020), and is also associated with greater yield stability (Li et al., 2021b; Wu et al., 2022). Intercropping also increases the diversity of crop residues compared with monocultures (Zhang et al., 2023a), and a key question remains whether and how intercropping may influence the decomposition of crop residues.

The main drivers of litter decomposition are litter type and the soil environment in which the litter decomposes (Powers et al., 2009; Guo et al., 2021). Differences in the chemical composition of plant litter may affect decomposition processes (Berg, 2014). For example, legume species have higher decomposition rates than non-legume species (Xu et al., 2017; da Silva et al., 2021). In addition to the importance of litter type, soil environmental conditions also play a key role in straw decomposition (Hättenschwiler et al., 2005; Chen et al., 2017a). For example, high soil water contents stimulate decomposition (Chen et al., 2017a). Intercropping influences soil water content (Yin et al., 2020), light transmittance (Li et al., 2021a), N dynamics (Chen et al., 2019) and soil enzyme activities (Curtright and Tiemann, 2021), which together influence the rate of decomposition of species-diverse straw mass. For example, intercropping enhances soil total N content, especially in low-fertility soils (Li et al., 2021b). However, it remains largely unknown how straw type and the soil environment in intercropping interact to influence straw decomposition.

Nitrogen addition to soils may also affect plant litter quality and thus influence decomposition processes, with N addition having positive (Vivanco and Austin, 2011; Li et al., 2017), negative (Song et al., 2019), or no effects on litter decomposition rates (Wang et al., 2019). Net N effects on litter decomposition depend on N fertilization rates and litter quality. For example, litter decomposition is inhibited by N additions when fertilizer N rates are high or when litter quality is low (e.g. high lignin content), whereas decomposition is stimulated when ambient N deposition is low and litter quality high (e.g. low lignin content) (Knorr et al., 2005). Here we focus on the effects of nitrogen fertilization on straw decomposition in intercropping systems.

There is considerable variation in the quality of plant litter returned to the soil (Cornwell et al., 2008) and many soil microbial communities are adapted to decompose local litter (Ayres et al., 2009). A growing number of studies show that litter decomposes faster in its habitat of origin (i.e. ‘home’) relative to some other location (i.e. ‘away’) (Veen et al., 2015; Li et al., 2017) and this is termed the ‘home-field advantage’ (HFA) effect (Ayres et al., 2009). The HFA effect occurs when the quality of a given litter type is well recognized by the decomposer community in the environment of the ‘home’ plot. The “substrate quality–matrix quality interaction” (SMI) hypothesis suggests that the strength of the HFA will be greater as the quality of specific plant litter and the decomposition environment become more and more divergent (Freschet et al., 2012). Using litter mass loss data from 125 reciprocal litter transplants across 35 studies, a meta-analysis found that there was considerable variation in the strength and direction (sometimes opposite to expectations) of the HFA effect (Veen et al., 2015). For example, some studies show accelerated decomposition in the home environmental conditions relative to away conditions (Ayres et al., 2009; Li et al., 2017), whereas other studies show similar or even reduced decomposition at home compared to away (Ayres et al., 2006; Gießelmann et al., 2011). However, our knowledge of the relative roles of litter quality and the soil environment on HFA effects of litter decomposition in intercropping remains limited.

Most studies consider the litter decomposition of single crop species but the litter layer usually consists of a mixture of litter materials from different plant species (Chen et al., 2018). Litter mixtures influence decomposition in two alternative ways, through additive or non-additive effects (Hättenschwiler et al., 2005; Chen et al., 2018). Additive effects do not involve interactions among straw materials from different species during decomposition, with no differences between observed and expected litter decomposition rates in mixtures based on species composition (Chen et al., 2017b). Non-additive effects include an antagonistic effect (slower decomposition in the mixture than expected) or a synergistic effect (faster decomposition in the mixture than expected) (Liu et al., 2020). Numerous studies show that non-additive effects (synergism or antagonism) seem to be more common than additive effects in the decomposition of litter mixtures (Hättenschwiler et al., 2005; Chen et al., 2018). The release of secondary metabolites from specific litter species leads to antagonistic non-additive effects, whereas synergistic mixing effects may occur due to nutrient transfer among litter species and suitable microenvironmental conditions can stimulate the decomposition of poor litter quality (Hättenschwiler et al., 2005). We hypothesize that mixtures of straw from legumes and maize have synergistic mixing effects during decomposition in maize/legume intercropping systems.

The current study aims to quantify how straw type, the soil environment and their combined effects may influence straw decomposition in widely practiced maize/legume intercropping systems. We hypothesize that 1) home-field advantages of straw decomposition depend on species identity and species combinations; and 2) there are synergistic mixing effects on straw decomposition in intercropping systems.

## 2 Materials and methods

### 2.1 Study site

The study was conducted at the China Agricultural University Lishu Experimental Station (43.3° N, 124.4° E) in Lishu county, Jilin province, northeast China from May 2020 to May 2021. The mean daily air temperature during the experiment was 8.2°C. The maximum air temperature was 28.5°C observed on 8 June 2020 and the minimum was -23.6°C on 7 January 2021 (**Figure S1**). The soil type is Vertisol and the texture of the soil is clay loam (23.9% sand, 45.2% silt, and 30.9% clay) at 0–20 cm depth. The surface soil (0–20 cm depth) organic matter content is 16.6 g kg^-1^, with a pH of 5.45, a total soil N content of 0.96 g kg^-1^, 18.9 mg of Olsen-P kg^-1^, and 137 mg of available K kg^-1^ at the start of the long-term experiment (2017).

### 2.2 Field experimental design

#### 2.2.1 Sources of straw materials

The decomposition study was carried out within a long-term intercropping field experiment (see Zhang et al., 2021 for more details) including two N level treatments and five cropping systems (**Figures S2**, **S3**). The study was therefore a two-factor complete randomized block design with three blocks. The first factor was two N levels (no N addition (N0) and N addition (N1) and the second factor was five cropping systems, namely monoculture maize (*Zea mays* L. cv. Xianyu No. 335), monoculture soybean (*Glycinemax* L. Merrill. Jiyu No. 47), monoculture peanut (*Arachis hypogaea* L. cv. Baisha No. 1016), maize/soybean intercropping, and maize/peanut intercropping (**Figure S3**). A total of 30 plots (2 N levels × 5 cropping systems × 3 blocks) were used in the decomposition study.

In the N-addition treatments, 80 kg N ha^-1^ were applied to the soybean and peanut monocultures as urea, 240 kg N ha^-1^ to the maize monoculture, and 160 kg N ha^-1^ to the two intercropping systems (**Table S1**). In addition, 52 kg P ha^-1^ (as superphosphate) were applied and 83 kg K ha^-1^ (as potassium sulphate) were also applied to each experimental plot. Three decomposition experiments were conducted to quantify litter and soil effects on straw decomposition and potential home field advantages (HFA). In October 2019 the stems and leaves of maize, soybean, and peanut from the monocultures and intercropping systems were collected randomly from the long-term experiment after harvest, and the straw materials were used in experiments I-III.

#### 2.2.2 Experiment I (different straw types decomposing in the same plot)

The litter quality effect on straw decomposition was quantified by decomposing different straw types in the same plot in experiment I (**Figures S3**, **S4**). Ten straw treatments were used from five cropping systems (maize, soybean, peanut, maize-soybean mixture, and maize-peanut mixture) and 2 N levels (5 × 2 = 10 straw treatments) in the long-term experiment. All straw materials were decomposed in three newly established monoculture maize plots adjacent to the long-term experiment (**Figures S3**, **S4**). Monoculture maize plots were selected to represent standard soil conditions because three-fifths of the plots contained maize. Each maize monoculture plot received 240 kg N ha^-1^, 52 kg P ha^-1^ (as superphosphate), and 83 kg K ha^-1^ (as potassium sulphate) fertilizers as in the long-term experiment (**Table S1**).

#### 2.2.3 Experiment II (same straw type decomposing in different plots)

The potential effects of the soil environment on straw decomposition were assessed by decomposing the same straw types in different plots in the long-term intercropping experiment (Experiment II, **Figures S2**–**S4**). The same straw (monoculture soybean straw from N1 fertilizer) was decomposed in 30 plots (5 cropping systems × 2 N levels × 3 blocks). The initial chemical quality trait (i.e. C/N ratio) of soybean was intermediate between maize and peanut, and soybean straw materials were therefore selected as the standard straw.

#### 2.2.4 Experiment III (straw types decomposing in their ‘home’ plots)

Potential straw type and soil environmental effects on straw decomposition were determined by decomposing each straw type in its ‘home’ plot in the long-term intercropping experiment (Experiment III, **Figures S2**-**S4**). The straw materials from 30 plots (5 cropping systems × 2 N levels × 3 blocks) were decomposed in their corresponding plots (‘home’ plots), i.e., the straw materials were returned to their original plots. The straw materials decomposed *in-situ* in experiment III (‘home’ plot), in contrast to experiments I and II which are considered as two control experiments (or ‘away’ plots) for decomposition.

### 2.3 Straw and soil sampling

After overwinter air-drying, all stem and leaf sample were clipped into 2-3 cm-long fragments in early May 2020 and oven-dried at 40°C for 72 h to constant mass. All straw samples were placed in 15 × 10 cm polyethylene litterbags (mesh size 180 μm) with 4 g dried straw. The actual situation of straw remaining in the field was simulated by mixing percentages of stems and leaves of the three monoculture crops calculated according to the biomass of the corresponding monocultures estimated from 2017 to 2019 at harvest. In this way the following combinations were obtained: monoculture maize straw (stem 53% + leaf 47%), monoculture soybean straw (stem 57% + leaf 43%) and monoculture peanut straw (stem 55% + leaf 45%). The percentage of mixed straw of both crops in the intercropping systems (maize/soybean and maize/peanut) was calculated by the biomass in their respective intercropping systems from 2017 to 2019 at harvest. We thus prepared: (a) maize-soybean mixture straw (maize 75% + soybean 25%), which was composed of stem 40% and leaf 35% of intercropping maize and stem 15% and leaf 11% of soybean, and (b) maize-peanut mixture straw (maize 81% + peanut 19%), which comprised stem 43% and leaf 38% of maize and stem 10% and leaf 9% of peanut.

The number of litterbags in experiment I was 150 (i.e. 5 straw types × 2 N levels × 1 soil condition × 5 retrievals × 3 blocks), in experiment II 150 (i.e. 1 straw type × 5 cropping systems × 2 N levels × 5 retrievals × 3 blocks), and in experiment III 150 (5 straw and soil combinations × 2 N levels × 5 retrievals × 3 blocks) (**Figure S4**). Thus, a total of 450 litterbags were buried in the soil at 10 cm depth in the field experiment on 27 May 2020. The litterbags were placed in the center of two crop rows in the monoculture and intercropping systems, respectively (**Figures S4**, **S5**). Litterbags from each plot were sampled after decomposition for 44, 74, 109, 136, and 341 days and oven-dried (60°C, 48 h). Soil samples (0-10 cm depth) were taken from each plot using a soil auger (5-cm-diameter) and soil water content was determined by oven-drying (105°C, 48 h) to constant mass. Straw materials were removed from the litterbags and each sample was washed and gently sieved through a 0.25-mm mesh to remove any adhering soil particles. The straw samples were then transferred to labeled paper envelopes and oven-dried (60°C, 48 h) to constant mass. The dry straw samples were then weighed and the straw mass loss was determined from the initial mass and the samples were retained for further analysis. Oven-dried straw samples prior to decomposition were ground with a ball mill for chemical analysis. The initial straw C and N concentrations were determined using a C/N analyzer (Vario Micro cube, Elementar, Lagenselbold, Germany). The concentrations of cellulose, hemicellulose and lignin were determined using an Ankom A200 fiber analyzer (Ankom Technology, Macedon, NY) based on a modified Van Soest method.

### 2.4 Calculations

Straw mass loss was calculated using the oven-dried weights at each retrieval (Chen et al., 2017a):


eqn 1 $$\text{Mass loss}=\left(1-\frac{M_{t}}{M_{0}}\right)\;\times \;100$$

where M_t_ is the dry mass of straw remaining in litter bags at each sampling time and M_0_ is the initial dry mass of straw.

Decay constants (*k*) were obtained assuming simple negative exponential decay (Olson, 1963):


eqn 2 $$\ln\left(\frac{M_{t}}{M_{0}}\right)=-kt$$

where M_t_ is the straw mass at time t and M_0_ is the initial straw mass. The *k* value can also indicate decomposition rate.

The contribution of litter effects and soil effects to the home-field advantage (HFA) was compared between experiments I-II (‘away’ plots) and III (‘home’ plots). Both experiments I and III used the same straw materials from two intercropping systems and two N levels of the long-term experiment, but a different plot environment was used for decomposition (‘home’ soil in experiment III vs. maize plots in experiment I), thus the soil effect on home-field advantage was assessed from the difference between experiments I and III. Both experiments II and III used the same plots with two intercropping systems and two N levels for decomposition in the long-term experiment, but used different straw types (4 straw treatments in experiment III *vs*. soybean straw in experiment II), thus the litter effect on home-field advantage was assessed from the difference between experiments II and III. The litter effect and soil effect on home-field advantage were calculated as follows (Li et al., 2017):


eqn 3 $$\text{Soil effect on HFA}=\text{ln}\left(\frac{k{\text{III}}_{\text{Tx}}}{k{\text{I}}_{\text{Tx}}}\right)$$

eqn 4 $$\text{Litter effect on HFA}=\text{ln}\left(\frac{k{\text{III}}_{\text{Tx}}}{k{\text{II}}_{\text{Tx}}}\right)$$

where Tx indicates the five types of straw in N0 and N1 treatments and *k*III, *k*II, and *k*I are the *k*-values of Tx treatment in experiments III, II, and I, respectively. Positive soil effects on home-field advantage (HFA) indicate that a specific straw decomposed faster in the ‘home’ soil (experiment III) than in the given soil (monoculture maize plot, experiment I), and positive litter effects on home-field advantage indicate that one straw type (experiment III) decomposed faster than the standard straw (soybean straw, experiment II) in the same plot (Li et al., 2017).

The relative mixture effect (RME) on straw mass loss at the end of decomposition was calculated as:


eqn 5 $$\text{RME}=\frac{\left(\text{O}-\text{E}\right)}{\text{E}}\times \;100\%$$

where O was the observed mass loss of straw mixture and E was the expected value based on the case of each component straw type decomposed separately (Wardle et al., 1997).

### 2.5 Statistical analysis

Generalized linear mixed-effects models (GLMMs) were used to test for potential effects of fertilizer N (N0 *vs*. N1), straw type or/and cropping system (maize, soybean, peanut, maize-soybean, and maize-peanut) and their interactions on straw mass loss and soil water content at 5 retrievals (**Table S2**). Nitrogen and straw type were included as fixed factors, and block and day were included as random effects (Bolker et al., 2009). Generalized linear mixed-effects models (GLMMs) were also used to test for potential effects of fertilizer N (N0 *vs*. N1), straw type or/and cropping system (maize, soybean, peanut, maize-soybean, and maize-peanut) and their interactions on initial straw quality (the concentrations of C, N, cellulose, hemicellulose and lignin, C/N ratio and lignin/N ratio), liter effect on HFA, soil effect on HFA, and relative mixture effect; N and straw type were included as fixed factors and the block was included as a random effect (**Table S2**). The R package ‘nlme’ was used for the linear-mixed effect models. Moreover, Pearson correlation analysis was used to test for potential relationships among initial straw quality and decomposition rate (k). Significant differences among treatments were determined by the *post-hoc* Tukey HSD test at *P*< 0.05. Furthermore, Student’s *t*-test was used to test the difference of HFA and RME from zero. We used R version 4.1.2 (R Core Team, 2022) for all statistical analysis.

## 3 Results

### 3.1 Effects of litter quality on straw mass loss (Experiment I)

**Figure 1:**
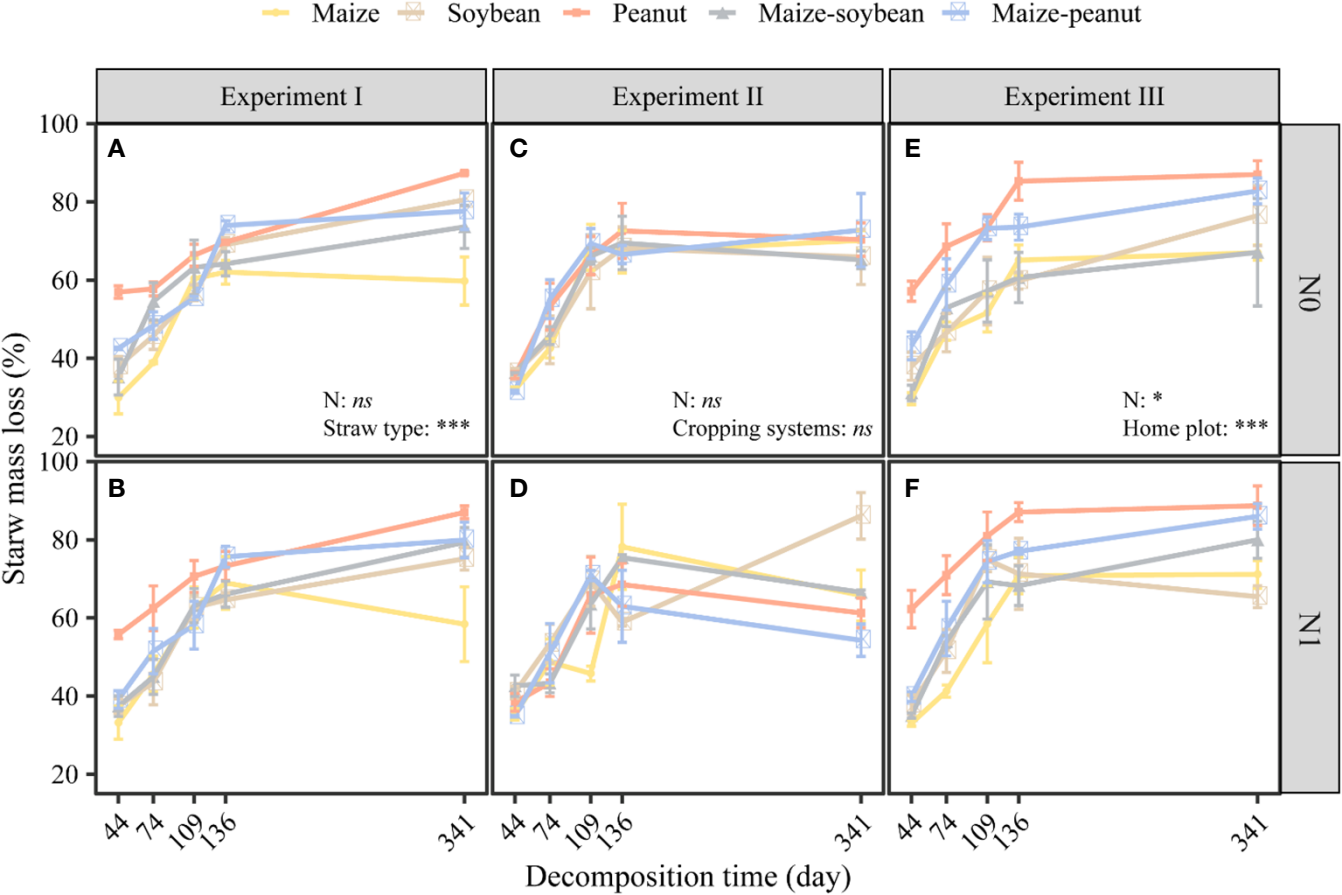
Effects of nitrogen addition and straw type on mass loss of different straws in the new monoculture maize plots **(A, B)**; effects of nitrogen addition and cropping system on straw mass loss of soybean straw **(C, D)**; and effects of nitrogen and cropping system on straw mass loss in the home plots **(E, F)**. In experiment I, different straw types decomposed in the same plot (new maize plots). In experiment II the same soybean straw decomposed in different plots. In experiment III, straw types from each plot decomposed in their home plot. *P < 0.05, *** *P* < 0.001, *ns*: not significant. Data are mean ± SE (*n*=3).

**Figure 2:**
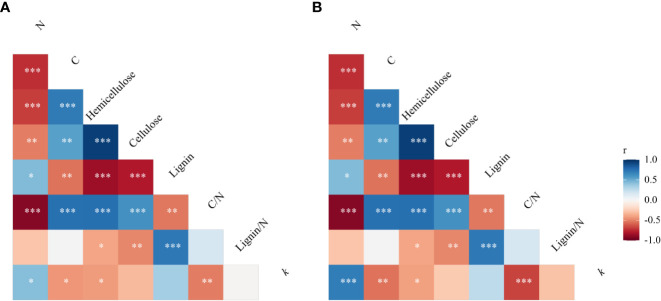
Pearson correlation matrix of potential relationships between decomposition rate (*k*) and initial straw quality (concentrations of C, N, cellulose, hemicellulose and lignin, C/N ratio and lignin/N ratio) in **(A)** experiment I and **(B)** experiment III. Associations are colored by direction of effect (blue, positive; red, negative), with associations significant (* *P*< 0.05, ** *P*< 0.01, *** *P*< 0.001).

Nitrogen did not significantly affect straw mass loss but straw type significantly affected straw mass loss in the same plot (maize plots) environment (*P*< 0.01). The mass loss values of maize, soybean, peanut, maize-soybean, and maize-peanut straw materials were 59, 77, 87, 76, and 78%, respectively, of the initial mass after 341 days of decomposition (**Figures 1A, B**). Peanut straw had the highest straw mass loss, which was significantly higher than mass loss of maize/peanut straw mixtures and maize/soybean straw mixtures and soybean, and maize had the lowest straw mass loss (*P*< 0.05) (**Figures 1A, B**). Nitrogen application significantly decreased the C/N ratios of straw while straw type significantly affected straw C/N ratios. The C/N ratio of maize was the highest (63.45 ± 5.60), followed by maize-soybean (58.77 ± 2.81), soybean (49.26 ± 2.53), and maize-peanut (47.71 ± 1.05). Peanut C/N ratio (21.69 ± 1.59) was significantly lower than those of other straw types (**Table 1**). The concentrations of cellulose and hemicellulose were highest in maize straw, but the lignin concentration was lower than in soybean and or peanut straw (**Table 1**). The initial C/N ratios as well as C and hemicellulose contents had a negative effect on decomposition but lignin and lignin/N ratio had no significant effects on the decomposition rate (*k*) under the same environmental conditions (**Figure 2A**).

**Table 1:** Initial quality traits of straw types (monoculture and mixture) from the different N addition treatments in experiments I – III.

N rate	Straw type	C (mg g^-1^)	N (mg g^-1^)	Cellulose (mg g^-1^)	Hemicellulose (mg g^-1^)	Lignin (mg g^-1^)	C/N	Lignin/N
N0	Maize	423.58 ± 2.57 aB	6.31 ± 0.65 bB	347.39 ± 3.22 aA	337.19 ± 3.32 aA	30.42 ± 3.3 cA	68.56± 6.82 aA	4.99± 0.86 bA
	Soybean	415.43 ± 1.54 aB	8.45 ± 0.29 bB	247.57 ± 5.59 bA	158.32 ± 9.55 cA	109.61 ± 7.5 aA	49.29± 1.87 bA	12.97± 0.75 aA
	Peanut	394.16 ± 11.28 bB	16.67 ± 0.43 aB	260.11 ± 13.59 bA	146.66 ± 15.69 dA	101.61 ± 8.68 bA	23.64± 0.26 cA	6.12± 0.65 bA
	Maize-soybean	420.78 ± 1.41 aB	6.98 ± 0.29 bB	325.01 ± 0.69 aA	305.4 ± 2.39 bA	51.14 ± 1.67 cA	60.52± 2.6 aA	7.33± 0.07 bA
	Maize-peanut	416.93 ± 4.82 aB	8.27 ± 0.2 bB	336.75 ± 1.64 aA	304.85 ± 2.03 bA	43.81 ± 2.24 cA	50.47± 1.46 bA	5.29± 0.15 bA
N1	Maize	436.5 ± 1.88 aA	7.56 ± 0.55 bA	343.69 ± 7 aA	338.04 ± 7.37 aA	40.02 ± 1.13 cA	58.34± 4.36 aB	5.37± 0.56 bA
	Soybean *	416.44 ± 5.79 aA	8.51 ± 0.42 bA	250.41 ± 7.78 bA	166.17 ± 7.22 cA	121.67 ± 6.8 aA	49.24± 3.2 bB	14.44± 1.5 aA
	Peanut	397.6 ± 6.78 bA	21.07 ± 3.18 aA	244.12 ± 25.18 bA	121.59 ± 10.18 dA	88.75 ± 13.79 bA	19.75± 2.91 cB	4.28± 0.65 bA
	Maize-soybean	428.99 ± 1.74 aA	7.57 ± 0.43 bA	313.98 ± 4.24 aA	300.47 ± 4.56 bA	52.22 ± 2.6 cA	57.01± 3.04 aB	6.97± 0.68 bA
	Maize-peanut	424.62 ± 0.7 aA	9.45 ± 0.14 bA	314.99 ± 4.61 aA	290.99 ± 4.36 bA	46.5 ± 4.8 cA	44.94± 0.65 bB	4.91± 0.43 bA
*P*-value								
N		0.049	0.040	0.136	0.171	0.546	0.021	0.750
Straw type		<.0001	<.0001	<.0001	<.0001	<.0001	<.0001	<.0001
N × Straw type		0.787	0.322	0.757	0.288	0.371	0.531	0.258

Means with different letters are significantly different (Tukey’s post hoc test; *P*< 0.05). Uppercase letters indicate differences between zero-N addition and N addition, and lowercase letters indicate differences among straw types. In experiment II the same straw (soybean from N1 treatment) decomposed in different plots, marked with an asterisk. Data are mean ± SE (*n* = 3).

### 3.2 Effects of cropping system on straw mass loss (Experiment II)

Nitrogen addition and cropping system did not significantly affect the mass loss of the same soybean straw materials (**Figures 1C, D**). Nitrogen significantly decreased soil water content but cropping system did not affect soil water content (**Figure S6**).

### 3.3 Effects of the ‘home’ plot on straw mass loss (Experiment III)

The mass loss of maize, soybean, peanut, maize-soybean and maize-peanut straw types was 66, 74, 80, 72, and 76% respectively, of the initial mass after 341 days of decomposition (**Figures 1E, F**). In general, peanut straw had significantly (*P*< 0.001) higher mass loss than maize, soybean, maize-soybean, or maize-peanut straw types during the experimental period (**Table S3**).

Nitrogen and cropping systems significantly affected the mass loss of straw in the ‘home’ plot (*P*< 0.05). Nitrogen fertilizer significantly increased the straw mass loss in ‘home’ plots. Peanut straw left to decompose in peanut monoculture plots had higher mass loss than other straw types including maize-peanut straw mixtures in maize/peanut intercropping systems. Maize-soybean straw mixtures, maize, and soybean had the lowest mass loss in their ‘home’ plots among the five straw types (**Figures 1E, F**). The initial C/N ratios, C and hemicellulose had a negative effect, but lignin and lignin/N ratio had no significant effects on straw mass loss in their ‘home’ plot (**Figure 2B**).

### 3.4 Effects of N addition and straw type on the home-field advantage effect

**Figure 3:**
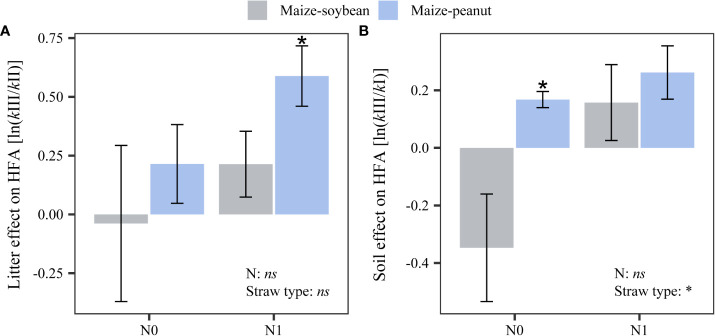
Effects of nitrogen addition and straw type on the home-field advantage effect *via* **(A)** litter type (‘litter effect’) and **(B)** soil environment (‘soil effect’). Asterisks above bars indicate significant differences between the home-field advantage effect and zero (*P*< 0.05). Statistical significance of the main effects of nitrogen application and straw type on HFA was also shown (*P< 0.05, *ns*: not significant).. Data are mean ± SE (*n*=3).

A significant litter type effect (‘litter effect’) was found in the home-field advantage (HFA) decomposition for maize-peanut straw mixture under nitrogen additions (0.59 ± 0.13; **Figure 3A**). This indicates that maize-peanut straw mixture decomposed faster than the standard straw (soybean straw) under higher N additions in the ‘home’ plot conditions. A positive ‘soil effect’ in the HFA decomposition for maize-peanut straw mixtures was found under zero N addition (0.17 ± 0.03), indicating that maize-peanut straw mixture decomposed faster in ‘home’ plots than in a given plot (maize plot condition) under zero N addition. The ‘soil effect’ in the HFA of maize-peanut straw mixtures was significantly higher than the HFA in maize-soybean straw mixtures (*P*< 0.05; **Figure 3B**).

### 3.5 Effects of N addition and straw type of relative mixing effects on litter decomposition

**Figure 4:**
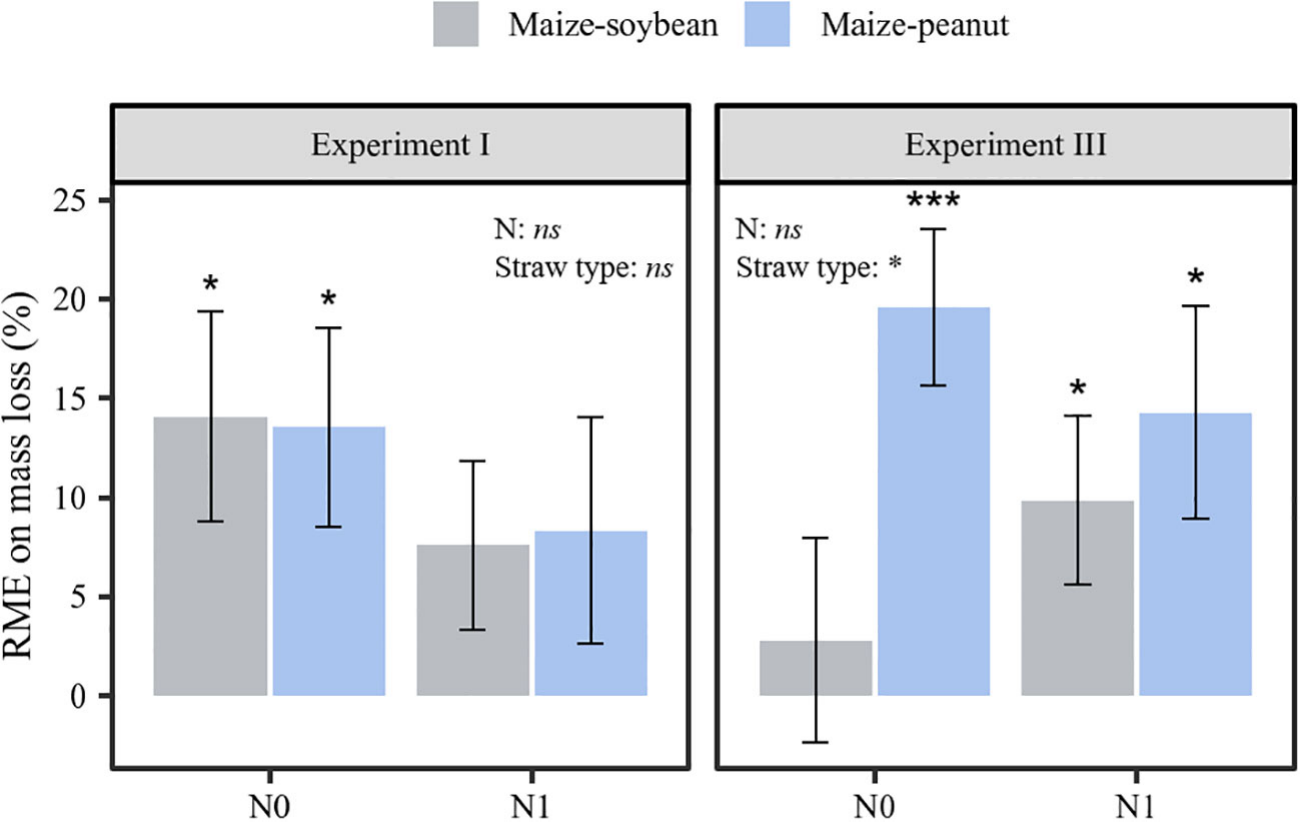
The relative mixing effects (RME) on straw mass loss in experiments I and III for maize-soybean and maize-peanut mixtures at N0 and N1 conditions. Data are mean ± SE (*n*=3). Asterisks above bars show significant differences between mean value and 0 (*P < 0.05, *** *P* < 0.001). Statistical significance of the main effects of nitrogen application and straw type on RME was also shown (*P < 0.05, *ns*: not significant).

After the five retrieval times of the litter bags were taken into consideration, the RME values of both maize-peanut (13.54 ± 5.02%) and maize-soybean mixture (14.05± 5.30%) decomposition were positive in the zero N-addition control in experiment I (*P*< 0.05; **Figure 4**). The magnitude of the RME of the maize-peanut mixture decomposition was significantly greater than that of maize-soybean mixtures in experiment III (*P*< 0.05; **Figure 4**). In addition, the RME of maize-peanut mixtures was positive in non N-addition (19.60± 3.96%) and N-addition treatments (14.28± 5.37%), whereas the RME of maize-soybean mixtures (9.84± 4.25%) was significantly positive only with nitrogen application in experiment III (*P*< 0.05; **Figure 4**).

## 4 Discussion

The results indicate that straw type had significant effects on the mass loss of straw in the same soil (i.e. maize plots in experiment I) and in the ‘home’ plot environment (experiment III), and this is mainly attributable to differences in straw quality (i.e. the C/N ratios). The mass loss of the same soybean straw materials was unaffected by the soil environment in the different cropping systems (experiment II). Nitrogen addition significantly increased straw mass loss in the ‘home’ plots (experiment III). Maize-peanut straw mixture decomposition showed higher occurrence and magnitude of ‘home-field advantage’ effects than mixed maize-soybean straw. Both maize/soybean and maize/peanut straw mixtures showed synergistic mixing effects (RME) on decomposition in both ‘home’ (experiment III) and ‘away’ plots (experiment I), while maize-peanut straw mixture decomposition had greater synergistic effects than maize-soybean straw mixture decomposition in the ‘home’ plots (experiment III).

### 4.1 The home-field advantage effects *via* straw quality and soil properties

The results here support our first hypothesis that home-field advantages (HFAs) of straw decomposition depend on species identity and on litter mass species combinations. A ‘litter identity effect’ of maize-peanut mixture straw on decomposition was found which was driven by increased nitrogen additions (**Figure 3A**). These findings suggest that the quality of maize-peanut mixture straw was significantly different from soybean straw, and the HFA effect occurred *via* litter quality. A ‘soil environment effect’ on the decomposition of maize/peanut mixture straw was also found in the unfertilized plots (**Figure 3B**). This suggests that the soil environment in maize/peanut intercropping is significantly different from that in the maize monoculture and that the HFA effect occurred because of ‘home’ soil properties.

There is increasing recognition that plants are associated with species-specific decomposer communities (McGuire and Treseder, 2010; Freschet et al., 2012). The HFA would likely occur when these decomposer communities are adapted to decompose local plant litter materials (Madritch and Lindroth, 2011; Osburn et al., 2022). There were no litter or soil effects associated with HFA in many other treatments. For example, we did not detect any HFA in maize and soybean straw materials decomposing in their ‘home’ plots (**Figure 3**), and these results support those from a worldwide meta-analysis in which HFA effects showed considerable variation in their strength and direction (Veen et al., 2015).

The results here indicate that straw type significantly affected straw mass loss during decomposition in the maize plot environment (experiment I) and in the ‘home’ plot conditions (experiment III). Different decomposition rates were also found in maize, soybean, and wheat straw materials (Xu et al., 2017). Peanut had the highest straw mass loss which was higher than maize/peanut straw mixtures, maize/soybean straw mixtures and soybean, and maize had the lowest straw mass loss among the five straw types (**Figures 1A, B**). Generally, residues with higher C/N ratios decompose more slowly than those with lower C/N ratios (Guo et al., 2021). Maize had the highest C/N ratio (63.4), followed by maize/soybean (58.8), soybean (49.3), and maize/peanut (47.7), and peanut (21.7) was significantly lower than other straw types (**Table 1**). The peanut straw had the highest mass loss during the period of decomposition whereas maize straw, soybean straw, and their mixture were more recalcitrant to decomposition. In addition, the current study indicates that the C/N ratio was a more important factor than lignin concentration in explaining the effects of straw quality on decomposition (**Figure 2**; **Table 1**). This result is consistent with those of a recent meta-analysis in which C/N ratio was a controlling factor in leaf litter decomposition across different biomes (Guo et al., 2021).

Nitrogen application did not affect straw mass loss in the ‘away’ plots (experiments I and II) but significantly increased soil water content and thus the straw mass loss in the ‘home’ plots (experiment III). Nitrogen and cropping systems did not significantly affect the mass loss of soybean straw materials (experiment II) (**Figures 1C, D**). These findings, together with those from experiments I-III, suggest that straw type was more important than the soil environment in affecting decomposition. The differences among litters were generally much larger than the effects of fertilization on litter decomposition (Hobbie, 2008). A recent meta-analysis also indicates that litter quality plays a more important role in driving leaf and fine root decomposition than soil and decomposers across biomes (Guo et al., 2021).

### 4.2 Maize-peanut, not maize-soybean, had home-field advantage in decomposition of straw mixtures in intercropping systems

The decomposition of maize-peanut straw mixtures was associated with a higher occurrence and magnitude of HFA effects than the decomposition of maize-soybean straw mixtures (**Figure 2**). The current results indicate that maize/peanut intercropping differed from maize and soybean monocultures in terms of straw quality and soil environment. This supports the “substrate quality-matrix quality interaction (SMI) hypothesis” in which HFA effects become larger when the quality of ‘home’ and ‘away’ litters become more dissimilar (Ayres et al., 2009; Freschet et al., 2012; Li et al., 2017). The C/N ratio of peanut straw was significantly lower than those of maize and soybean straw materials (**Table 1**) thus promoting greater HFA effects in maize-peanut straw decomposition than in maize-soybean straw decomposition through enhanced litter quality. Two meta-analysis studies also found that HFA effects increased with the initial litter quality (N content and C/N ratio) (Zhang et al., 2013; Veen et al., 2015). In addition, HFA can be affected by dissimilarity in the decomposer community and tends to become stronger when plant communities are more dissimilar (Veen et al., 2015). The maize/peanut intercropping system can significantly modify soil microbial community composition and the dominant microbial species relative to maize and peanut monocultures (Li et al., 2016), thus promoting HFAs in maize-peanut straw decomposition *via* changes in the soil environment. The current results indicate that maize-peanut residue retention in their home field may be an alternative method of straw return practice in maize/peanut intercropping.

### 4.3 The relative mixing effects on maize-legume mixtures

The present study partly supports the second hypothesis that synergistic mixing effects of straw decomposition occur in intercropping systems. The RME associated with maize-peanut and maize-soybean mixture decomposition was positive under zero N-addition in new maize monoculture plots (experiment I). Moreover, the RME of maize-peanut mixture decomposition was significantly positive under two nitrogen levels; and the RME of maize-soybean mixtures was significantly positive under N addition in their ‘home’ plots (experiment III) (**Figure 4**). These findings suggest a synergistic effect of maize-soybean and maize-peanut litter mixtures on decomposition. Previous studies indicate that litter mixtures may show more non-additivity (synergism or antagonism) rather than additivity in their decomposition rates (Hättenschwiler et al., 2005; Hou and Lü, 2020). The synergistic effects of decomposition of maize-soybean and maize-peanut litter mixtures may be due to nutrient transfer (e.g. nitrogen, phosphorus) from high-quality legumes to low-quality maize straw or the complementarity effects of soil fauna and decomposers (Schimel and Hättenschwiler, 2007; Luan et al., 2022).

The RME of maize-peanut straw mixture decomposition was significantly higher than that of maize-soybean straw in their ‘home’ plots (*P*< 0.05, **Figure 4**). Across agroforestry systems it has been shown that non-additive effects were most pronounced when a high-N-concentration litter was mixed with a low-N-concentration litter (Wang et al., 2014). RME values increased in magnitude with increasing dissimilarity in the traits of litter mixtures, with positive effects related to trait dissimilarity in the case of nutrient traits and negative effects related to trait dissimilarity in recalcitrance traits (Canessa et al., 2022). The lower C/N ratio of peanut straw than of soybean straw materials (**Table 1**) thus promoted higher RME values in maize-peanut straw decomposition than in maize-soybean straw decomposition through enhanced litter quality. The current results partly support the higher yield of maize intercropped with peanut than with soybean in the same long-term intercropping experiment due to increased nutrient release and transfer (Zhang et al., 2021; Yang et al., 2022; Zhang et al., 2023b).

The overall straw mass loss in the current experiments was about 70% of the initial mass by day 136, and there was relatively little further straw decomposition subsequently (from days 136 to 341). This latter effect may be explained by the recalcitrant components left in this stage and the dry and cold winters in northeast China. The average temperature was 20.7°C and the cumulative precipitation was 514 mm at the early stages of decomposition (days 1 to 136). In contrast, the average temperature was -2.8°C and the cumulative precipitation was 36.6 mm at the later stages of decomposition (**Figure S1**).

Furthermore, the proportion of different litter species has been 1:1 in most previous studies. Here, to simulate the actual situation of straw remaining in the field, the percentage of the legume was nearly 20% in mixed straw while the RME effect of the maize-legume was also observed, and the higher percentage of legume may lead to greater synergistic mixing effects. Maize residue retention is a recommended practice in China and globally, and the current results indicate that mixing maize and legume residues can improve straw management practices by promoting straw decomposition and nutrient release.

## 5 Conclusion

Straw type significantly affected the straw mass loss in the same plots (i.e. maize plots) and in the ‘home’ plot environment, and this is largely explained by the different straw C/N ratios (litter quality). Neither N addition nor cropping system significantly affected the mass loss of soybean straw (experiment II). Straw type explained more the variation in straw decomposition than did the soil environment. Nitrogen addition did not affect the same straw mass loss in other locations but increased the straw mass loss in the ‘home’ plots. In addition, maize-peanut straw mixtures had a higher occurrence and magnitude of HFA effects than maize-soybean straw mixture materials. There was a synergistic mixing effect of maize-peanut straw mixture and maize-soybean straw mixture decomposition in both ‘home’ and ‘away’ plots. Maize-peanut straw mixtures showed a significantly higher relative mixing effect than maize-soybean straw mixture decomposition in their ‘home’ plots (**Figure S7**). The results suggest that *in situ* maize/peanut residue retention is to be recommended due to its higher straw decomposition and nutrient release rates in intercropping systems in temperate regions.

## Data availability statement

The original contributions presented in the study are included in the article/**Supplementary Material**. Further inquiries can be directed to the corresponding author.

## Author contributions

SS and W-PZ designed the research. SS, W-PZ, YS, Y-HD, HY and S-XR collected the data. SS and W-PZ analyzed the data. SS drafted the paper, and LL, DF, PC, W-PZ and SS contributed substantially to the revisions. All authors contributed to the article and approved the submitted version.
