# Supplementary material for: Maize/peanut intercropping has greater synergistic effects and home-field advantages than maize/soybean on straw decomposition
Source: Front Plant Sci. 2023 Mar 3;14:1100842. doi: 10.3389/fpls.2023.1100842 (PMC10020597; doi:10.3389/fpls.2023.1100842)
Supplement: Supplementary file 1 [file DataSheet_1.docx]

Supplementary Material

## Supplementary Figures


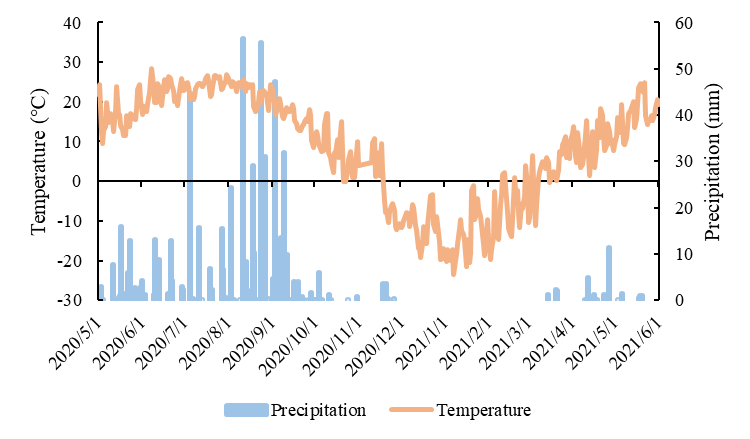


**Fig. S1.** Air temperature and precipitation in the field from 1 May 2020 to 1 June 2021.

**
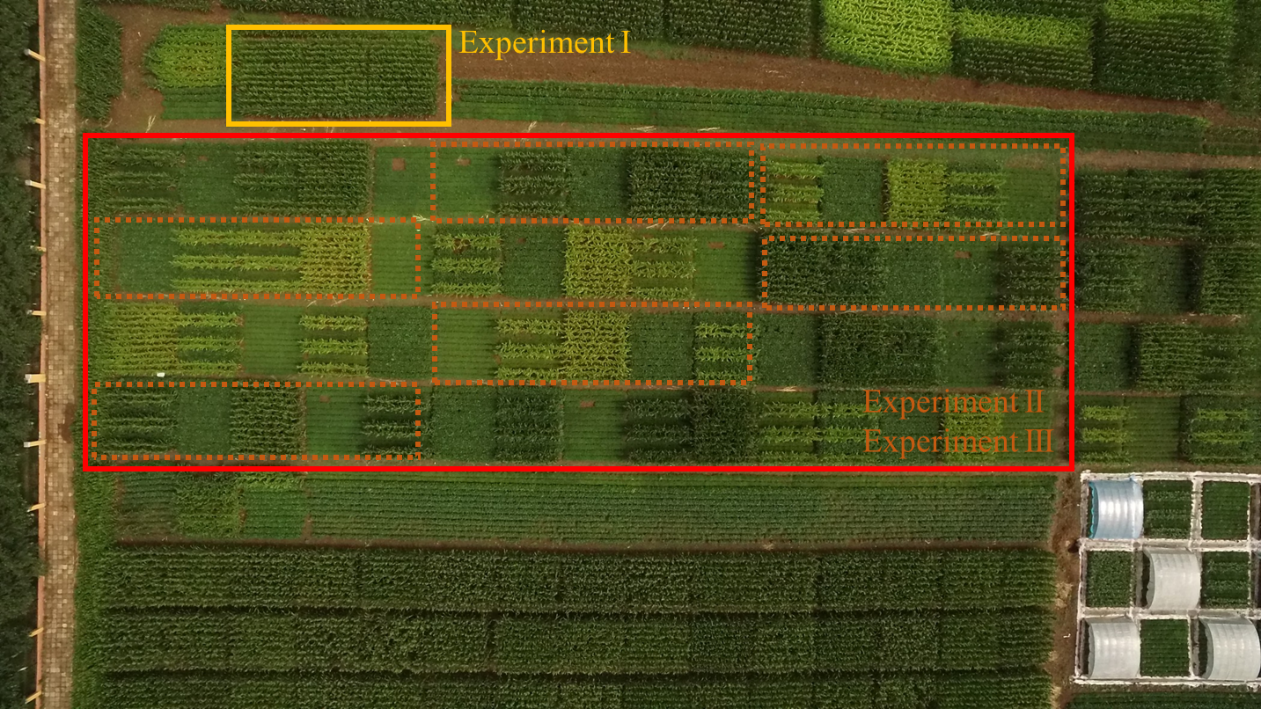
**

**Fig. S2.** Aerial photograph showing the layout of the long-term intercropping experiment and experiments I-III in 2020. The yellow frame represents experiment I, which is adjacent to the long-term field experiment (red solid line frame). The red dotted line frames indicate the plots of decomposition experiments II and III.


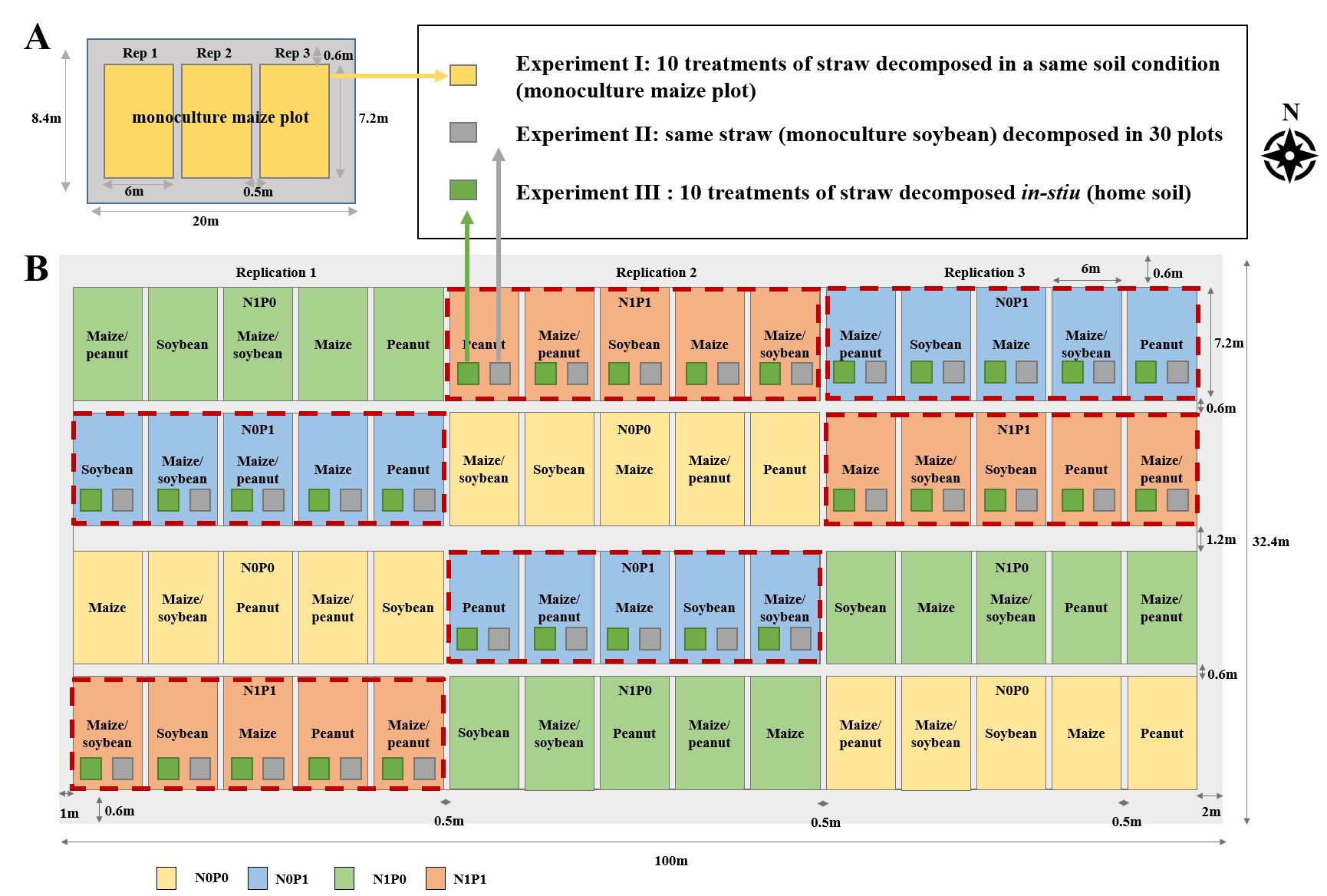


**Fig. S3.** Layout of the long-term maize/legume intercropping experiment and experiments I-III; 2 N levels and 5 cropping systems from the long-term experiment were selected for the decomposition study.

(A) Experiment I was used to quantify the litter quality effects on decomposition: five types (maize, soybean, peanut, maize-soybean, and maize-peanut mixture) of straw from two nitrogen treatments decomposed on the same maize plot;

(B) Experiment II was used to quantify the soil environmental effects on decomposition: soybean straw decomposed on different plots (5 cropping systems and 2 N levels); and experiment III: the straw types were returned to where they originated, and decomposed on their ‘home’ plot in the long-term experiment. Both experiments II and III used the same plots with 5 cropping systems and 2 N levels for decomposition in the long-term experiment. The red dashed frames illustrate the selected plots for experiment II and experiment Ⅲ treatments (*n* = 3).


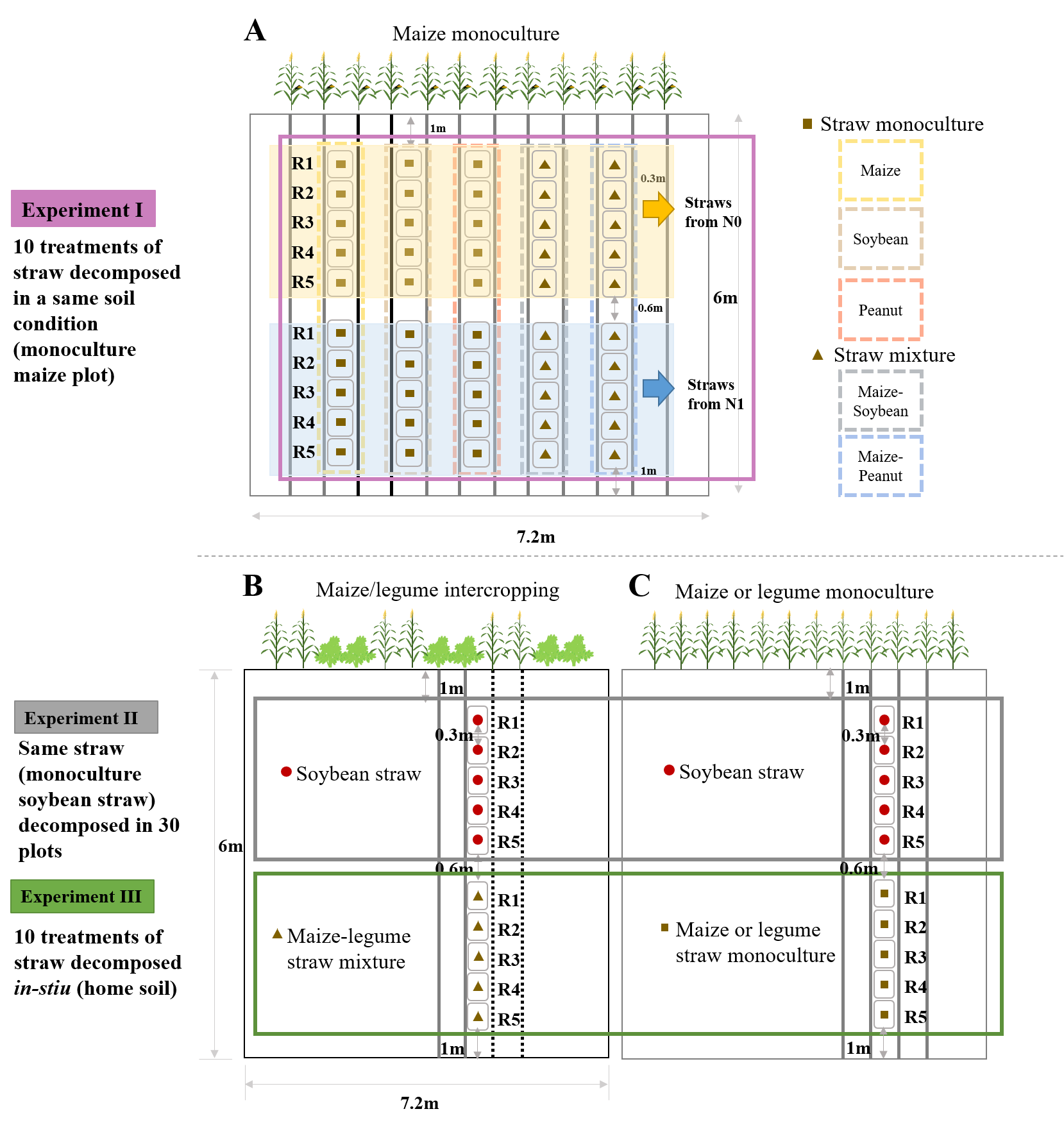


**Fig. S4.** Diagrammatic representation of the position of litterbags in (A) experiment I, (B) experiment II and (C) experiment Ⅲ in 2020. The number of litterbags in experiment I was 150 (i.e. 5 straw types × 2 N levels × 1 soil condition × 5 retrievals × 3 blocks), five types of straw (maize, soybean, peanut, maize-soybean, maize-peanut as indicated by the different colored line frames) from N0 and N1 treatments (indicated by yellow and blue shaded areas) decomposed in 3 new monoculture maize plots. The number of litterbags in experiment II was 150 (i.e. 1 straw type × 5 cropping systems × 2 N levels × 5 retrievals × 3 blocks), and in experiment III 150 (5 straw and soil combinations × 2 N levels × 5 retrievals × 3 blocks). Both experiments II and III used the same plots with 5 cropping systems and 2 N levels for decomposition in the long-term experiment. R1-R5 denote corresponding retrieval at days 44, 74, 109, 136, and 341.


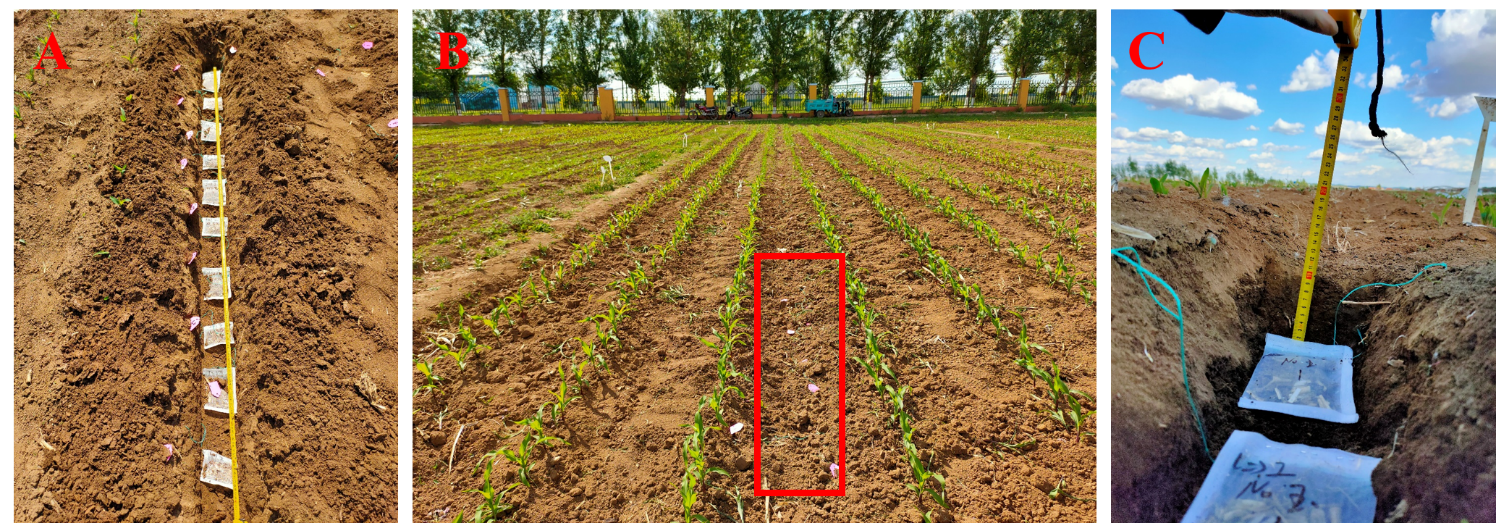


**Fig. S5.** The litterbags were placed in the center of two crop rows in the monoculture and intercropping systems (A and B), respectively, and buried in the soil at 10 cm depth (C).


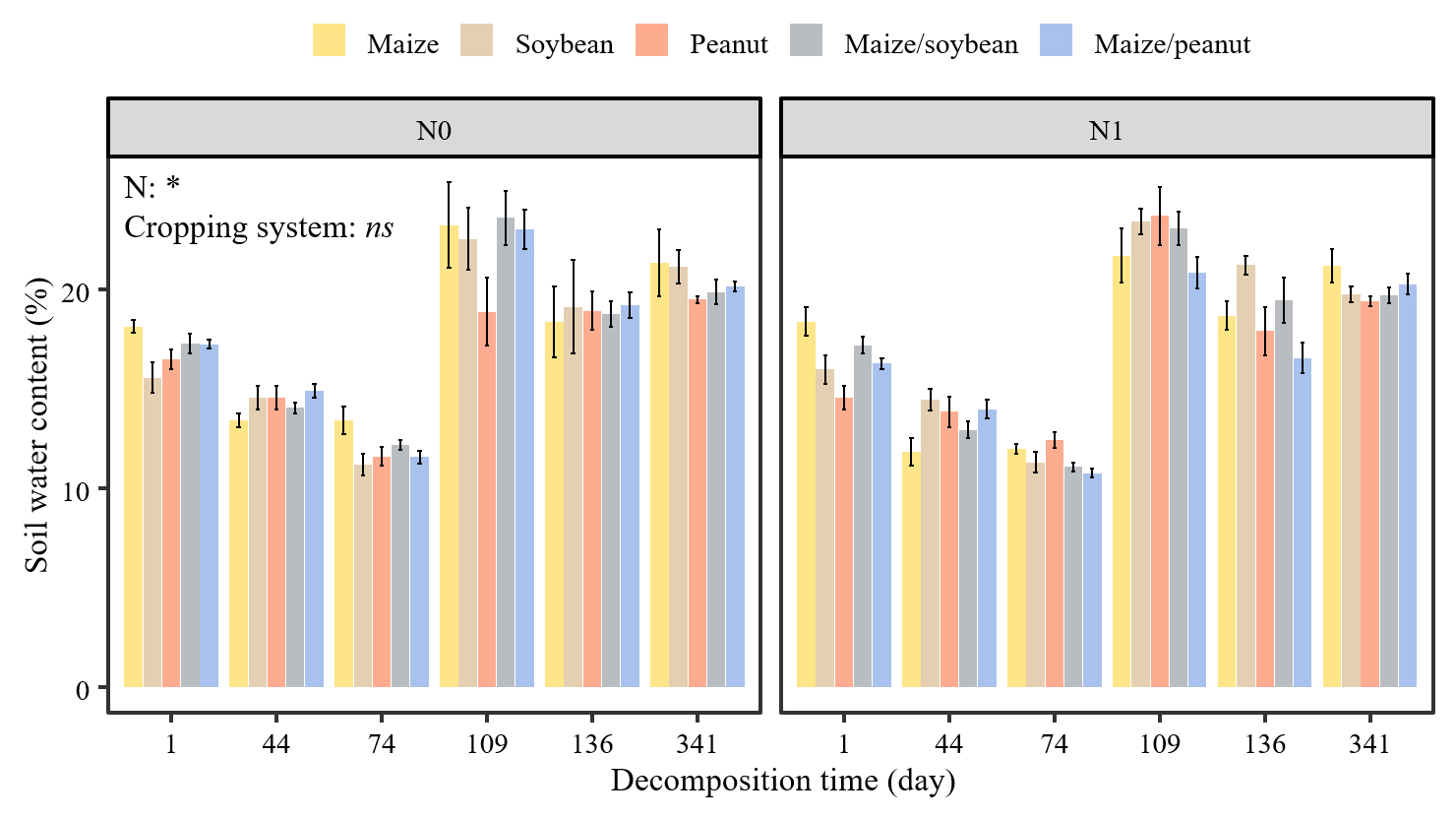


**Fig. S6.** Soil water content in maize, soybean and peanut monocultures, maize/soybean intercropping, and maize/peanut intercropping under N0 and N1 conditions (experiments II and III). **P* < 0.05, *ns*: nonsignificant. Data are mean ± SE (*n*=3).


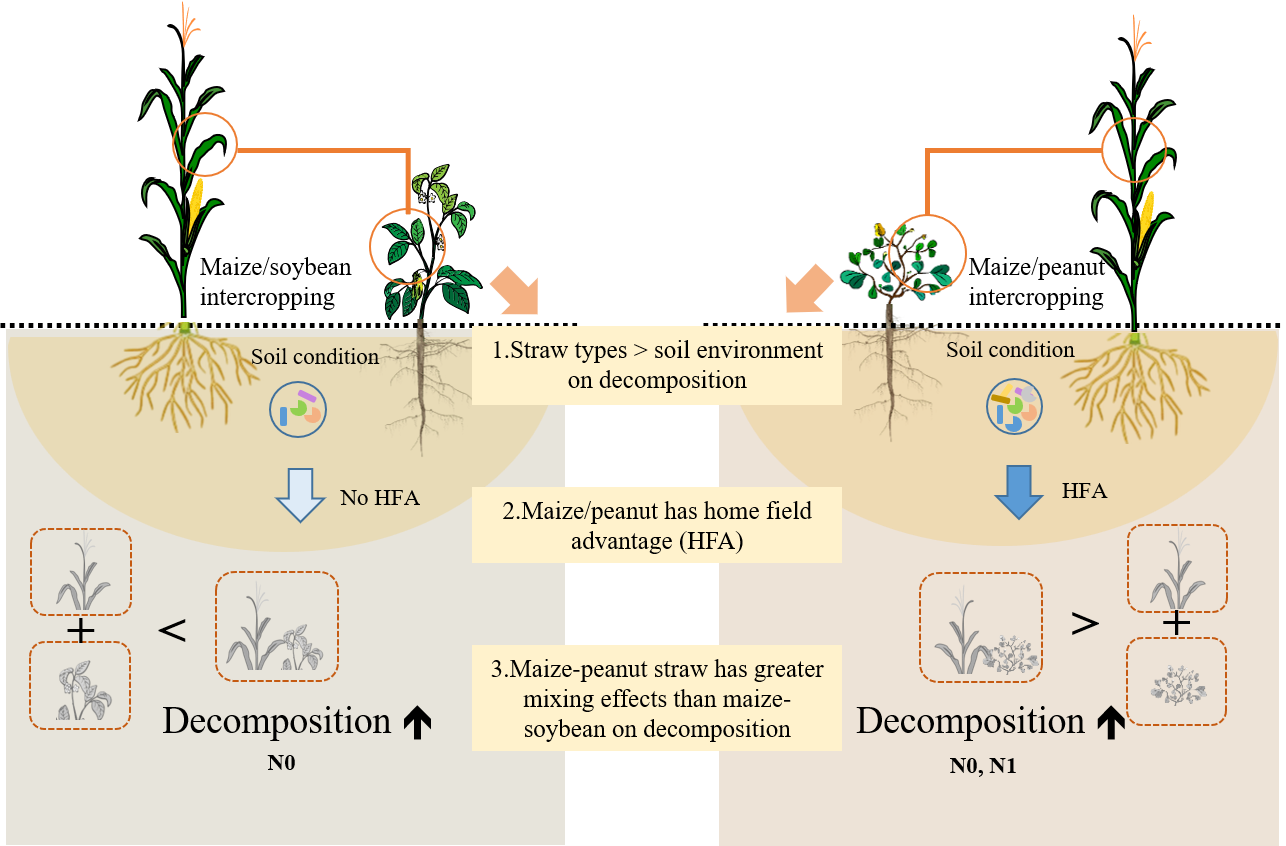


**Fig. S7.** Conceptual diagram of how straw type, the soil environment and their combined effects influence straw decomposition in maize/legume intercropping systems.

## Supplementary Tables

**Table S1** Experimental treatments applied to both intercrops and monocrops

| Cropping system | N treatment | Total N rate  (N kg ha^-1^) | Basal N rate  (N kg ha^-1^) | Topdressing N rate  (N kg ha^-1^) | |
| --- | --- | --- | --- | --- | --- |
|  |  |  |  | Elongation stage | Pre-tasseling stage |
| Maize/legume intercropping | N0 | 0 | 0 | 0 | 0 |
|  | N1 | 160 | 80 | 40 | 40 |
| Monoculture maize | N0 | 0 | 0 | 0 | 0 |
|  | N1 | 240 | 100 | 60 | 80 |
| Monoculture legume | N0 | 0 | 0 | - | - |
|  | N1 | 80 | 80 | - | - |

**Table S2** List of mixed effects models fitted to the data. *Day*_i_ and *α*_i_ are random block effects, *ε*_i_ is a residual error, *α*_i_ and *ε*_i_ are assumed to be normally distributed with constant variances.

| Model | Explanatory variable | Equation |
| --- | --- | --- |
| 1 | Straw mass loss, k value at experiment I | （*Straw mass loss, k value*)_i_ = β0 + β_1_**N*_i_ +β_2_**Straw type*_i_ +  β3 **N*_i_ **Straw type*_i_ + *Day*_i_ + *α*_i_ + *ε*_i_ |
| 2 | Straw mass loss, k value at experiment II | （*Straw mass loss, k value*)_i_ = β0 + β_1_**N*_i_ +β_2_**Cropping system*_i_+  β3 **N*_i_ **Cropping system*_i_ + *Day*_i_ + *α*_i_ + *ε*_i_ |
| 3 | Straw mass loss, k value at experiment III | （*Straw mass loss, k value*)_i_ = β0 + β_1_**N*_i_ +β_2_**Home plot*_i_ +  β3 **N*_i_ **Home plot*_i_ + *Day*_i_ + *α*_i_ + *ε*_i_ |
| 4 | Soil water content | （*Soil water content*)_i_ = β0 + β_1_**N*_i_ +β_2_**Cropping system*_i_  +β3 **N*_i_ **Cropping system*_i_ + *Day*_i_ + *α*_i_ + *ε*_i_ |
| 5 | Litter effect | （*Litter effect*)_i_ =β0 + β_1_**N*_i_ +β_2_**Straw type*_i_ +  β3 **N*_i_ **Straw type*_i_+ *α*_i_ + *ε*_i_ |
| 6 | Soil effect | （*Soil effect*)_i_ = β0 + β_1_**N*_i_ +β_2_**Cropping system*_i_+  β3 **N*_i_ **Cropping system*_i_ + *α*_i_ + *ε*_i_ |
| 7 | Initial quality, Relative mixture effect | （Initial quality, *Relative mixture effect*)_i_ =β0 + β_1_**N*_i_ +  β_2_**Straw type*_i_ +β3 **N*_i_ **Straw type*_i_ + *α*_i_ + *ε*_i_ |

Initial straw quality denotes the concentrations of C, N, cellulose, hemicellulose and lignin, C/N ratio and lignin/N ratio of straw types.

**Table S3** Straw mass loss of decomposition experiments in the different N addition treatments

| N rate | Straw type / Cropping system | Experiment I ^†^ | Experiment II ^‡^ | Experiment III ^¶^ |
| --- | --- | --- | --- | --- |
| N0 | Maize | 50.2±15.05 c | 57.03±16.7 a | 53.67±13.92 c |
|  | Soybean | 54.54±14.54 bc | 56.78±15.8 a | 56.86±15.72 c |
|  | Peanut | 66.21±10.7 a | 60.46±14.84 a | 75.52±12.44 a |
|  | Maize-soybean | 57.04±15.05 b | 58.03±13.88 a | 55.01±16.86 c |
|  | Maize-peanut | 59.57±14.92 b | 61.02±16.1 a | 67.98±14.64 b |
| N1 | Maize | 53.42±15.92 c | 54.75±18.33 a | 54.93±17.71 c |
|  | Soybean | 56.66±15.36 bc | 61.95±17.76 a | 60.2±16.14 c |
|  | Peanut | 69.82±12.19 a | 54.55±14.88 a | 77.97±12.57 a |
|  | Maize-soybean | 58.16±16.6 b | 58.27±14.41 a | 61.27±17.86 c |
|  | Maize-peanut | 60.86±17.06 b | 54.79±15.07 a | 66.9±18.1 b |
| *P*-value | |  |  |  |
| N | | 0.131 | 0.652 | 0.015 |
| Straw type (or Cropping system) | | <0.001 | 0.795 | <0.001 |
| N × Straw type (or Cropping system) | | 0.783 | 0.108 | 0.381 |

Means with different letters are significantly different (Tukey’s post hoc test; *P* < 0.05). Straw mass loss is shown as mean with standard error (n = 15). Lowercase letters indicate differences among straw types and/or cropping systems.

^†^ The difference between different straw types was compared in experiment I, as different straw types were decomposed in the same plot (maize plots).

^‡^ The difference between different cropping systems was compared in experiment II, as the same straw was decomposed in different plots.

^¶^ The difference between different cropping systems and straw type combinations was compared in experiment III, as straw types from each plot were decomposed in their home plots.
